# Genetic Diversity of Equid Herpesvirus 5 in Temporal Samples from Mares and Their Foals at Three Polish National Studs

**DOI:** 10.3390/ijms26178298

**Published:** 2025-08-27

**Authors:** Karol Stasiak, Magdalena Dunowska, Jerzy Rola

**Affiliations:** 1 Department of Virology and Viral Animal Diseases, National Veterinary Research Institute, Al. Partyzantow 57, 24-100 Pulawy, Poland; jrola@piwet.pulawy.pl; 2 School of Veterinary Science, Massey University, Palmerston North 4442, New Zealand; m.dunowska@massey.ac.nz

**Keywords:** EHV-5, equine viruses, sequence analysis, haplotype network, gammaherpesviruses

## Abstract

Equid herpesvirus 5 (EHV-5) comprises a group of heterogeneous viruses with a worldwide distribution. Primary infection typically occurs early in life, which is followed by latency and periodic recrudescence of the virus. The aim of this study was to determine the genetic variation of EHV-5 in individual animals over time and to determine the dynamics of EHV-5 spread among selected mare–foal pairs at three horse studs. The partial glycoprotein B (*gB*) gene was amplified from archival nasal swab samples. Sequences from 3–5 clones from each PCR product were compared using identity matrix, phylogeny, and median-joining haplotype networks. Overall, 328 clones were sequenced from long PCR products amplified from 84 EHV-5 PCR-positive swabs. The sequences were heterogeneous (89.4% to 100% nucleotide identity). The EHV-5 sequences from mares and their foals most often clustered separately, although similar EHV-5 sequences from the same mare–foal pair were also recovered. For some animals, the EHV-5 sequences from multiple sampling times clustered together, while sequences from other animals were distributed throughout the networks. Clones from the same PCR product were most often similar to each other, but divergent clones from the same PCR product were also apparent. In conclusion, the foals were likely to acquire EHV-5 infection from sources other than their dams, but some exchange of EHV-5 between mares and their foals also occurred. Some foals likely acquired EHV-5 from a single source, while others from multiple sources. These data contribute to our understanding of EHV-5 variability and the dynamics of infection in individual horses.

## 1. Introduction

Equid herpesvirus type 5 (EHV-5) is one of the five herpesviruses that infect horses [1]. It is classified in the family *Herpesviridae*, subfamily *Gammaherpesvirinae*, and genus *Percavirus* [2]. EHV-5 shares a considerable sequence similarity with another equid gammaherpesvirus, EHV-2, and is also similar to human herpesvirus 4 (Epstein–Barr virus, EBV) [3]. EHV-5 infection has been detected in horses worldwide at frequencies between 14% and 100%, depending on the sample type, the detection method, the sampling strategy, and the age of the population sampled [4,5,6,7,8,9]. In most studies, the frequency of EHV-5 detection was higher in young horses compared to older ones [4,10,11,12,13]. Following primary infection, which often occurs within the first year of life, the virus establishes a lifelong latent infection in B lymphocytes [14].

The exact role that this virus plays in the development of disease is poorly understood [15]. The virus has been frequently detected in horses without an overt clinical disease [6,13]. However, it has also been putatively linked to respiratory disease and poor performance in young horses [1,15,16], as well as multinodular pulmonary fibrosis (EMPF) in the aged ones [17,18].

Only one full genomic sequence of EHV-5 is publicly available [19]. Typical for a herpesvirus genome, the genome of EHV-5 consists of genes conserved among different herpesviruses, interspersed with species-specific genes and non-coding regions. Most sequence data available for EHV-5 comprises partial glycoprotein B (*gB*) gene sequences, as this genomic region has been commonly used in phylogenetic analysis of herpesviral genomes [20]. Glycoprotein B is highly conserved and has homologues among all members of the family *Herpesviridae* [21]. It is essential for virus entry to the cell and cell-to-cell spread [21,22]. The *gB* of EHV-5 is a disulfate-linked heterodimer that forms an integral part of the viral envelope [23,24].

Both EHV-2 and EHV-5 comprise heterogeneous populations of viruses, although the impact of this genetic heterogeneity on the biology of these viruses remains unclear [25]. Results of previous studies suggest that the genome of EHV-5 may be slightly less variable than that of EHV-2, primarily based on the analysis of the *gB* gene [26,27]. In two recent studies of gammaherpesviruses detected from horses in Poland, the *gB* nucleotide identities ranged between 89.9% and 100% for EHV-5 [13] and between 82.4% and 100% for EHV-2 [6].

In a previous study, we showed that each of 76 mares and their foals that were followed monthly at three Polish national studs were positive for EHV-5 on at least one sampling occasion [12]. The aim of the current study was to investigate the variability of EHV-5 obtained from selected mares and foals from each of the three studs over time in order to determine the genetic variation in individual horses over time. Specifically, we hoped to gain some insights into the dynamics of EHV-5 spread among selected horses, including determining whether repeated detection of EHV-5 over several months represented reactivation of a latent virus, reinfection, or chronic infection with prolonged shedding.

## 2. Results

### 2.1. Phylogenetic Analysis

Overall, 328 clones were sequenced from long PCR products amplified from 84 EHV-5 PCR-positive swabs. EHV-5 sequences from each stud were distributed throughout the phylogenetic tree, indicating that several different genotypes of EHV-5 circulated at each stud (Figure S1). Overall, the EHV-5 sequences from the mares from Studs I, II, and III showed 94.5–100%, 90.1–100%, and 89.6–100% identity to each other, respectively. The EHV-5 sequences from foals were 90.6–100%, 90.4–99.8%, and 91.3–100% identical to each other at Studs I, II, and III, respectively (Figures S2–S4, Tables S1–S3). The overall identity of all the sequences from the same mare/foal pair showed a similar range to that observed between sequences from individual animals over time: 89.8–100%, 90.1–100%, and 89.4–100% for Studs I, II, and III, respectively (Table S4).

Clones from one PCR product did not always cluster together. For example, three clones from the PCR product from a November sampling of F4 from Stud II (c2, c3, and c4, green rectangles) clustered together with two clones from the October sampling of M4 (c3 and c5, green squares) in cluster 1E. The remaining clones from F4 from the November sampling were closely related to the cluster of sequences from that foal from other sampling times (Jul, Aug, and Sep) within cluster 1B (c5) and to several sequences from M4 from various sampling times (Jul, Aug, Sep, and Oct) within Group 3.

### 2.2. Haplotype Network Analysis

**Figure 1:**
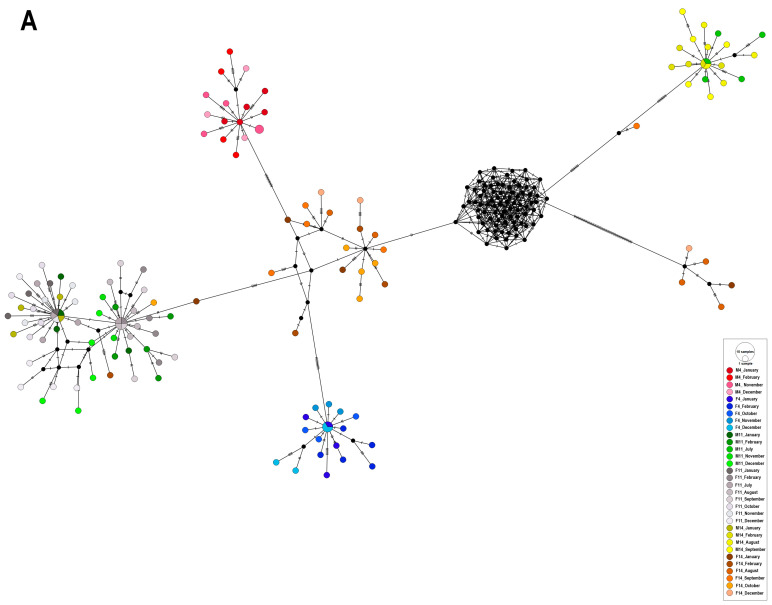

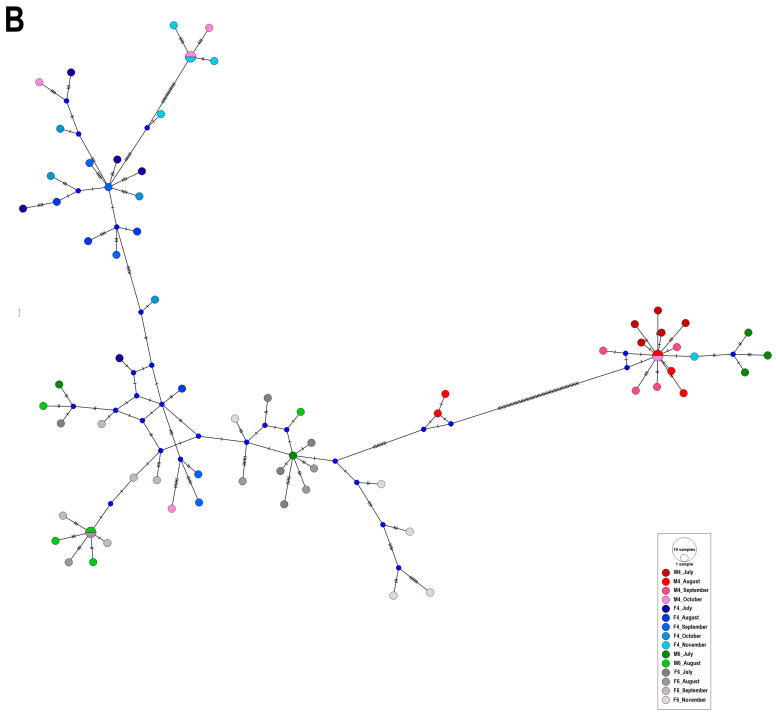

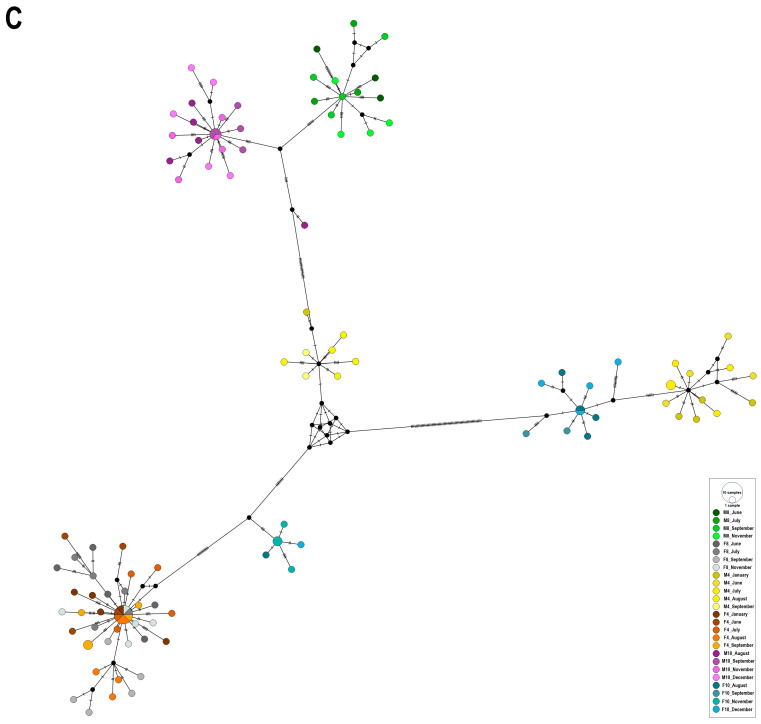
Median-joining haplotype network based on partial *gB* sequences of EHV-5 (concatenated and trimmed to 1245 nt) from Stud I (**A**) (*n* = 141), Stud II (**B**) (*n* = 72), and Stud III (**C**) (*n* = 115). The number of nucleotide substitutions between haplotypes is represented by ticks on branches. Nodes are scaled based on the number of representative sequences and coloured by individual sample, with shades of the same colour indicating samples from the same horse (F) or mare (M) at different sampling times. Small closed black circles indicate inferred nodes that are not represented among sequences included in the network.

The relationships between the EHV-5 *gB* sequences observed in the median-joining haplotype networks were similar for all three studs (Table 1). There was some structure in the networks when these were coloured by individual sample (Figure 1), with the strongest differentiation between sequences from different samples visible for Stud III (PhiST = 0.87, *p* < 0.001). This suggests that most of the variability between sequences from each horse/foal occurred between samplings, indicating that clones obtained from the same PCR product were more likely to be similar to each other than to clones obtained from other PCR products, irrespective of the source of the latter.

There was some structure visible in the network when sequences were coloured by individual horse/foal (Table 1). This was strongest for Stud III, where the variation between EHV-5 from different horses/foals (63.9%) contributed more to the overall variation than the variation between EHV-5 sequences from individual animals (36.9%, PhiST = 0.64, *p* < 0.001). For Studs I and II, the variability between the EHV-5 *gB* sequences from the same animals was slightly higher than that from different animals (PhiST = 0.42 − 0.44, *p* < 0.001, Table 1). The identical results were obtained when the age of the animal sampled (mare versus foal) was considered as a variable (Table 1).

At all three studs, the EHV-5 *gB* sequences from each mare/foal pair showed considerable variation when considered over the entire sampling period, with only 6% to 16% variability attributed to differences between populations (PhiST < 0.2, *p* < 0.05, Table 1).

Similarly, there was no structure in the network when the sequences were coloured by age of the sampled animals (mare/foal) for studs I and II, with 56% to 58% of variability attributed to variation within populations (PhiST < 0.44, *p* < 001). However, for stud III, 64% of the variability was attributed to differences between populations (PhiST = 0.64, *p* < 0.001), indicating that sequences from foals and mares differed at that stud. Similar results were obtained when the networks were coloured by individual horse/foal for Studs I and II, with most variability occurring within populations (among samples from each individual horse/foal throughout the study period). At Stud III, the variability of EHV-5 *gB* sequences from different horses contributed more (64%) to the overall variability than the variability of sequences from each individual horse (36%) over the study period (PhiST = 0.637, *p* < 0.01, Table 1).

Finally, there was no correlation between the sampling month and haplotype, with haplotypes from different samplings clustering together and haplotypes from the same sampling distributed throughout the networks (PhiST < 0.1 for all three studs, Table 1). The *p*-value was not significant for Stud II, likely reflecting the smallest number of EHV-5 *gB* sequences analysed from this stud (Table S2).

## 3. Discussion

The current study complements previously published data on the epidemiology of gammaherpesviruses among horses from three different Polish studs [12]. Specifically, we hoped to find out whether repeated isolation of EHV-5 from nasal secretions of sampled foals and mares over several months represented reactivation of latent infection, persistent infection, or reinfection. We also hoped to elucidate whether dams are the most common source of infection to their foals.

Consistent with the results reported previously [13], the EHV-5 *gB* sequences reported in the current study were heterogeneous, with identity scores similar to those reported previously (89.9% to 100%) when larger numbers of horses from 13 different Polish studs were sampled. This suggests that the variability between the EHV-5 sequences selected for the current study was representative of the EHV-5 heterogeneity expected to be found in various populations of horses. Consistent with previously reported results [13], while some phylogenetic clusters contained sequences from one stud only (e.g., clusters A and C within Group 1, Figure S1), most clusters contained sequences from more than one stud, indicating that similar EHV-5 viruses were circulating at the three studs, despite their geographical separation.

Clones from individual PCR products were overall more likely to be similar to each other than to clones from a different PCR product (Table 1), with many showing >99% nucleotide identity to each other (Tables S1–S3). However, over half (55/105, 52%) of the PCR products had at least one clone that differed from the rest, with nucleotide identities ranging from 89.8% to 99%. Since we used a high-fidelity polymerase for amplification of the long PCR products, the difference of >1% in the 1.3 kpb sequence of individual clones from the same PCR product is unlikely to represent an amplification error and more likely to reflect amplification of the EHV-5 DNA from different genotypes of EHV-5 present in the sample. Similarly to our results, Back et al. [28] reported that 7/18 horses sampled twice, one year apart, were infected with more than one genotype of EHV-5 on at least one sampling occasion. In contrast, Dunowska et al. [29] reported that all clones obtained from each PCR product amplified from 15/17 EHV-5 isolates had identical *Bfa 1* restriction patterns, even though the patterns differed between the EHV-5 obtained from different horses. The clones from only two isolates, derived from the same clinical sample, showed three distinct restriction patterns. The discrepancies between the results of that study and the current study likely reflect differences in the methodology used to detect variation (restriction patterns versus sequencing) and differences in the starting material (cell culture isolates versus nasal swabs). Altogether, our data suggest that co-infection of one horse with different genotypes of EHV-5 is common. These could represent concurrent infection with EHV-5 from different sources, concurrent recrudescence of different latent viruses, or a mixture of the two. Alternatively, it is also possible that the variability in EHV-5 obtained from the same clinical sample reflects the ongoing within-host adaptation of the virus, driven by the individual genetic makeup and immune response. The concept of viral quasispecies, historically applied to RNA viruses only, has been recently extended to DNA viruses, including herpesviruses [30,31], with considerable between- and within-host diversity of herpes simplex type 2 observed in samples from a neonatal unit [32].

Multiple EHV-5 sequences obtained at different sampling times from some horses/foals in the current study appeared closely related, with little change over several months of sampling. Examples include F4 (blue) and M14 (yellow) from Stud I (Figure 1A), or M8 (green), M10 (purple), F8 (grey), and F4 (orange/brown) from Stud III (Figure 1C). This finding is consistent with previous reports. In one study, the *gB* gene from six EHV-5 isolates obtained from samples collected from a single foal over a 9-month period showed an identical *Bfa 1* restriction pattern [29]. Two of the viruses originated from nasal swabs collected when the foal was 5 and 13 months old, while the remaining isolates were co-cultured from the foal’s peripheral blood leucocytes (PBL) when it was 9, 11, 12 and 13 months old, suggesting that repeated isolation of EHV-5 from this foal’s nasal swabs 8 months apart represented reactivation of the latent virus rather than re-infection. This conclusion was strengthened by the fact that EHV-5 isolated from other horses included in the same study showed horse-specific *Bfa 1* restriction patterns, including two identical isolates from PBL samples collected one month apart from another foal. While these conclusions were based on a fairly crude method of comparison (restriction enzyme digestion of a PCR product), Back and others [28] also reported detection of the same genotype of EHV-5 in each of 7 out of 9 horses that were sampled twice, a year apart, based on next-generation sequencing of *gB* amplicons [28].

Despite the detection of similar genotypes from some of the sampled foals/horses over time, the AMOVA analyses demonstrated that EHV-5 *gB* sequences from the same animal were more likely to be similar to each other than to other sequences over the entire study period only for Stud III, with approximately 60% of the overall variability contributed to differences in the EHV-5 *gB* from the same animal for Studs I and II (Table 1). This was most likely a reflection of the fact that some horses were infected with more than one genotype of EHV-5. Some of those heterogeneous genotypes (positioned in different parts of the networks) appeared transient. However, there was typically at least one genotype that was consistently detected in samples collected from each horse/foal over the entire sampling period. This can be deduced from the high upper limit of “between-samplings” nucleotide identities for all horses/foals sampled (99.8% to 100%, Table S4). It could be argued that the persisting genotype represents the genotype that is most adapted to an individual horse/foal or the one that established latency. If so, repeated detection of such a genotype is likely to represent recrudescence of latent infection. This is particularly likely in situations where highly similar EHV-5 clones were detected at two or more sampling times that were interspersed with samplings at which EHV-5 was not detected from the nasal swab samples, which occurred for some mares but not foals (Table 2).

Similar EHV-5 sequences would be expected following periodic recrudescence of a latent virus, while more diverse EHV-5 sequences may be detected following reinfection with one of the viruses circulating in the population. If so, it could be concluded that both recrudescence and reinfection contributed to the persistence of EHV-5 in the sampled populations. Alternatively, these results may reflect concurrent reactivation of more than one genetically different EHV-5 in each horse. In this scenario, the variability of EHV-5 sequences observed would depend on the variability of the latent EHV-5. Despite considerable research interest in equine gammaherpesviruses over the past 20 years or so, the molecular mechanisms governing the latency and recrudescence of these viruses remain poorly understood. As such, the genetic diversity of latent viruses remains undetermined, and whether or not some are more likely to recrudesce than others remains unknown.

Only one previous study investigated the variability of EHV-5 in samples collected from mares and their foals over several months [26]. In that study, some EHV-5 sequences from 12 mare–foal pairs were similar to each other, while others differed, suggesting varied sources of EHV-5 infection in foals [26]. This was in contrast to EHV-2, which appeared to be transmitted predominantly between mares and their foals in the same study when the foals were 1 month of age and between foals later, when the foals were older. In the current study, both foals from Stud II were positive for EHV-5 at the first sampling time, making it difficult to determine the timing and source of EHV-5 infection in those foals. However, all three foals from Stud III and two foals (F4 and F14) in Stud I were negative for EHV-5 at the first sampling time (Table 2). The EHV-5 clones from these five foals at the next sampling occasion were all different from the EHV-5 clones of their dams, suggesting that the source of the initial infection for these five foals was not their dams. Foal F14 from Stud I was likely to acquire EHV-5 from various sources, as the EHV-5 *gB* sequences from that foal were distributed throughout the network (Figure 1A, orange), while foal F4 (Figure 1A, blue) from Stud I was likely infected with EHV-5 from a single source (unsampled in the current study). Similarly, none of the EHV-5 sequences from mares and foals at Stud III clustered together (Figure 1C). Sequences from various sampling occasions of F4 (orange) and F8 (grey) all clustered together in the bottom left of the network (Figure 1C), suggesting exchange of EHV-5 between these two foals or a common source of infection. All sequences from M8 (green) formed one cluster at the top of the network, while sequences from M4 (yellow) formed two separate clusters, suggesting two different sources of EHV-5 infection for this mare. Similarly, the EHV-5 *gB* sequences from F10 (blue) formed two clusters, separately from sequences from its dam, which formed a cluster at the top of the network (purple).

Although the exact source of EHV-5 could not be determined for most of the foals included in the current study, the fact that EHV-5 *gB* sequences from foals most commonly clustered separately from sequences of their dams and some of them clustered together suggests that foals are more likely to acquire EHV-5 from other foals than from their dams. We have previously shown that foals typically shed higher quantities of EHV-5 than the mares and that the EHV-5 sequences from the foals were often more similar to sequences from other foals than to sequences from their dams, which supports this conclusion [12,13]. The foals and mares at Studs II and III were kept in pairs in individual stalls, with daily turnouts as a group in a shared paddock. The transmission between foals could have occurred through direct contact or via fomites, such as water troughs, feeding buckets, and others. The M11/F11 and M14/F14 pairs from Stud I were kept with other mares and foals in the open barn, while M4/F4 were kept at the open reserve in conditions closely matching the natural environment of the Konik Polski breed.

Interestingly, the clusters containing the haplotypes of EHV-5 *gB* from M11/F11 and M14/F14 seemed more mixed than the haplotypes from M4 (red) and F4 (blue) (Figure 1A), possibly reflecting more opportunities for exchange of EHV-5 between horses in the barn (e.g., via fomites) compared with the open reserve. A big cluster of closely related sequences at the left in the network (Figure 1A) contained a mix of clones from M11 (green) and F11 (grey). There were also some haplotypes from F14 (orange) and M14 (yellow) in the same cluster, suggesting that the two mares and their foals acquired EHV-5 from a similar source. However, most of the haplotypes from M14 were visible in the right corner of the network, where they clustered together with another group of haplotypes from M11 (green). Hence, it appears that transmission of different genotypes of EHV-5 may have occurred at different sampling times between M11 and F11 and between M11 and M14, or that these horses were exposed to two different (unsampled) sources of the virus during the study period.

Despite the overall separation between the EHV-5 *gB* sequences from mares and foals, some clones from selected mares and foals were similar to one another. For example, several clones from July–August samplings of M6/F6 from Stud II all clustered together in group 1C. However, since two clones from the July–August samplings of F4 from that stud were also in the same group, the EHV-5 could have been transmitted between all three horses. Similarly, individual clones from some mare–foal pairs from later samplings also clustered together, suggesting transmission of these viruses between mares and their foals. Examples include two clones from the October sampling of M4 (c5 and c3) and three clones from the November sampling of F4 (c2, c3, and c4) from Stud II, which clustered together in Group 1E, or several clones from various sampling times of M11/F11 from Stud I, which clustered in Group 1A (Figure S1 and Figure 1A). This suggests that the EHV-5 viruses were occasionally exchanged between foals and their dams during the study period.

The main limitation of this study is the relatively small number of horses and foals followed at each stud, which precludes generalisation of the findings to a larger population. Inclusion of all mares and foals from a single stud or management group would have provided a clearer picture of the patterns of EHV-5 transmission within the group. An earlier start of the sampling, ideally within a few days after foaling, and more frequent sample collection would have also provided more information on the initial sources of infection for the foals and routes of transmission of the viruses within a management group. Some transient infections and genotype changes may have been missed at monthly samplings. Finally, it was difficult to establish based on the available data if repeated detection of EHV-5 with similar *gB* sequences represents chronic active infection or frequent recrudescence of EHV-5, particularly for foals, as once EHV-5 was detected in the nasal swab of each foal, that foal was continuously positive for EHV-5 in subsequent samplings (Table 2). Collection of blood samples for the detection of the latent virus, in addition to more frequent sampling, would have helped to shed some light on this issue. Despite these limitations, this is only the second study in which EHV-5 from a group of mares and their foals followed over a period of time was analysed. Hence, our data provide a valuable contribution to the current state of knowledge of the genomic variability and dynamics of EHV-5 infection within a group of horses and within individual animals.

## 4. Materials and Methods

### 4.1. Source of Samples

DNA extracted from nasal swab samples obtained during a previous study [12] was used as a starting material. Briefly, healthy mare–foal pairs from three national horse studs were sampled monthly over a period of six to nine months. Breeds included Polish Konik horse (Stud I), Arabian (Stud II), and Thoroughbred (Stud III). The management of horses at each stud, sampling dates, and results have been described in the previous publication [12]. Two to three mare/foal pairs were selected from each stud for the current study. The factors used in the selection were the frequency of EHV-5 detection over the study period and the Cq values at each sampling. The results of EHV-5 detection from samples collected from the selected animals are summarised in Table 2. The first sampling was carried out when the foals included in the study were 3 to 5, 3 to 4, and 1 to 2 months of age for studs I, II, and III, respectively [12].

### 4.2. Conventional PCR Assays and Cloning

Viral DNA from selected (*n* = 84) EHV-5-positive swabs was used as a template in a conventional PCR assay targeting a fragment of the *gB* gene, with the expected product size of 1339 bp. Two (stud II) or three (studs I and III) mare/foal pairs were selected for the analyses. PCR reactions to amplify the *gB* gene of EHV-5 were performed as described previously [13]. Each PCR reaction contained 600 nM of each primer (forward: 5′-CCAACACAGAAGACAAGGAG-3′; reverse: 5′-CACGGTGATACAGTCAGAGA-3′), 1 x buffer (Sigma-Aldrich, St. Louis, MO, USA), 0.2 mM deoxynucleotide mix (Sigma-Aldrich), 0.5 µL JumpStart AccuTaq LA DNA Polymerase (Sigma-Aldrich), and 2 µL of template DNA in a total volume of 25 µL. Amplifications were performed in a Biometra Thermocycler (Biometra, Göttingen, Germany) using the following cycling conditions: 5 min of initial denaturation at 94 °C, followed by 35 cycles of denaturation (1 min at 94 °C), primer annealing (1 min at 59.5 °C), elongation (1 min at 72 °C), and a final elongation step (7 min at 72 °C). Amplified products were subjected to electrophoresis through a 1.5% GelRed stained agarose gel, purified with GeneJET PCR Purification Kit (Thermo Scientific, Carlsbad, CA, USA) and ligated into TOPO TA Cloning Kit for Sequencing (Thermo Fisher Scientific, Carlsbad, CA, USA), according to the manufacturer’s instructions. Ligated products were chemically transformed into *Escherichia coli* JM109 Competent Cells (Promega Corporation, Madison, WI, USA). Plasmid DNA was isolated from up to five insert-positive clones using PureLink™ Quick Plasmid Miniprep Kit (Thermo Fisher Scientific, Vilnius, Lithuania) according to the manufacturer’s instructions and sequenced with the same primers used for amplification using BigDye^®^ Terminator version 3.1 (Applied Biosystems, Vilnius, Lithuania) on a 3730xl DNA Analyzer at the Genomed (Warsaw, Poland).

### 4.3. Sequence Analyses

The sequences obtained from each recombinant *E. coli* colony were trimmed to an identical length (1245 bp) and assembled using BioEdit software v.7.2.5. Only sequences that generated high-quality data were included in the analysis. The consensus sequences were aligned with the reference sequence of EHV-5. 2-141/67 (GenBank accession number NC_026421.1) using the MUSCLE algorithm from the Molecular Evolutionary Genetics Analysis software package, version 10 (MEGAX) [33]. Identity among sequences analysed in the study was calculated using the “Sequence Identity Matrix” formula in BioEdit and illustrated as heatmaps using a Sequence Demarcation Tool Version 1.3 (SDTv1.3) [34]. Phylogenetic tree was constructed using the maximum likelihood method with 1000 bootstrap replicates using the K2+G substitution model in MEGAX. All nucleotide sequences from one stud were used as an input file to generate a haplotype list in the DnaSP software (version 6.12.03). The haplotypes representative of the EHV-5 sequences were exported in nexus format with trait blocks added to represent individual sample, individual animal, mare–foal pair, source (mare/foal), and sampling (month). The median-joining haplotype networks were drawn using default parameters in PopART version 1.7 (available from http://popart.maths.otago.ac.nz, accessed on 30 May 2025). Analysis of molecular variance (AMOVA within PopART) was carried out to test for correlation between the population genetic structure of the EHV-5 sequences and selected traits. The strength of correlation was represented by a PhiST value, with 0 indicating no correlation and 1 indicating strong correlation. The corresponding *p*-values were generated by reference to 1000 random permutations of the input data.

### 4.4. GenBank Accession Numbers

The nucleotide sequences described in this study were submitted to GenBank under the following accession numbers: PV650935–PV651262.

## 5. Conclusions

In summary, we have shown that EHV-5 comprises a heterogenous group of viruses. Both mares and foals can be infected with more than one genotype of EHV-5. Some genotypes seem transient, while others persist in individual horses long-term, most likely indicating the establishment of latency with periodic recrudescence. The foals are likely to acquire EHV-5 infection from sources other than their dams, but some exchange of EHV-5 between mares and their foals also occurs. These data contribute to our understanding of EHV-5 variability and the dynamics of infection in individual horses. Many questions regarding EHV-5 interactions with its equine host remain unanswered and should be addressed in future studies.

## Figures and Tables

**Table 1:** Analysis of molecular variance (AMOVA) results indicating the strength of correlation between population genetic structure within Polish equid herpesvirus 5 (EHV-5) and selected traits based on the partial sequence of *glycoprotein B*.

Test/Breed	Variation Within Populations	Variation Between Populations	PhiPT	*p* ^1^
**Individual sample**				
Stud I	32.6%	67.4%	0.67	<0.001
Stud II	37.3%	62.7%	0.63	<0.001
Stud III	13.1%	86.9%	0.87	<0.001
**Individual animal**				
Stud I	55.6%	44.4%	0.44	<0.001
Stud II	58.3%	41.7%	0.42	<0.001
Stud III	36.1%	63.9%	0.64	<0.001
**Mare–foal pair**				
Stud I ^2^	84.3%	15.7%	0.16	<0.001
Stud II ^3^	93.6%	6.4%	0.06	0.03
Stud III ^4^	86.3%	13.7%	0.14	<0.001
**Source of the virus (mare/foal)**				
Stud I	55.6%	44.4%	0.44	<0.001
Stud II	58.3%	41.7%	0.42	<0.001
Stud III	36.1%	63.9%	0.64	<0.001
**Sampling (month)**				
Stud I	93.2	6.8	0.07	0.003
Stud II	96.8	3.2	0.03	0.154
Stud III	92.6	7.4	0.07	0.012

^1^ probability that the random value > observed PhiST. ^2^ Three pairs + foals without mares; ^3^ Two pairs + foals without mares; ^4^ Three pairs.

**Table 2:** The summary of EHV-5 DNA detection results at different sampling times for mares (M) and foals (F) included in the current study. More details related to these data, including concurrent detection of EHV-2, virus load, and ages of the animals, can be found in Table S1 of the previous publication [12]. Samples positive for EHV-5 are labelled “+”; samples negative for EHV-5 are labelled “−”; months when the samples were not collected are labelled “nd”.

Animal	Sampling Month									
	May	Jun	Jul	Aug	Sep	Oct	Nov	Dec	Jan	Feb
Stud I										
M4	nd	nd	+	+	−	−	+	+	+	+
F4	nd	nd	−	−	−	+	+	+	+	+
M11	nd	nd	+	−	−	−	+	+	+	+
F11	nd	nd	+	+	+	+	+	+	+	+
M14	nd	nd	−	−	−	−	+	+	−	−
F14	nd	nd	−	+	+	+	+	+	+	+
Stud II										
M4	nd	+	+	+	+	+	+	+	nd	nd
F4	nd	+	+	+	+	+	+	+	nd	nd
M6	nd	−	+	+	+	−	−	−	nd	nd
F6	nd	+	+	+	+	+	+	+	nd	nd
Stud III										
M1	+	+	−	+	nd	+	+	nd	+	nd
F1	−	−	−	+	nd	+	+	nd	+	nd
M4	+	+	+	+	nd	+	+	nd	+	nd
F4	−	+	+	+	nd	+	+	nd	+	nd
M8	−	+	+	+	nd	+	+	nd	−	nd
F8	−	+	+	+	nd	+	+	nd	+	nd
M10	+	+	+	+	nd	+	+	nd	+	nd
F10	−	−	−	+	nd	+	+	nd	+	nd

## Data Availability

The nucleotide sequences described in this study were submitted to GenBank under the following accession numbers: PV650935–PV651262.

## Supplementary Materials

The following supporting information can be downloaded at https://www.mdpi.com/article/10.3390/ijms26178298/s1.
