# Supplementary material for: Genetic Diversity of Equid Herpesvirus 5 in Temporal Samples from Mares and Their Foals at Three Polish National Studs
Source: Int J Mol Sci. 2025 Aug 27;26(17):8298. doi: 10.3390/ijms26178298 (PMC12428152; doi:10.3390/ijms26178298)
Supplement: Supplementary file 1 [file ijms-26-08298-s001.zip › Supplementary_figures_captions.pdf]

Figure S1. Phylogenetic tree of equid herpesvirus 5 (EHV-5) based on 1,245 bp fragment from gB gene. The evolutionary history was inferred by using the Maximum Likelihood method and Kimura 2-parameter model. The tree with the highest log likelihood (-8436.34) is shown. The percentage of trees in which the associated taxa clustered together is shown next to the branches. Initial tree(s) for the heuristic search were obtained automatically by applying Neighbor-Join and BioNJ algorithms to a matrix of pairwise distances estimated using the Maximum Composite Likelihood (MCL) approach, and then selecting the topology with superior log likelihood value. A discrete Gamma distribution was used to model evolutionary rate differences among sites (5 categories (+G, parameter = 0.3375)). The tree is drawn to scale, with branch lengths measured in the number of substitutions per site. This analysis involved 328 nucleotide sequences. All positions with less than 90% site coverage were eliminated, i.e., fewer than 10% alignment gaps, missing data, and ambiguous bases were allowed at any position (partial deletion option). There were a total of 1196 positions in the final dataset. Evolutionary analyses were conducted in MEGA X. The Polish EHV-5 sequences obtained in the current study are labelled EHV5_stud number_month of sampling_horse ID number_clone number. Samples from the same stud are labelled with triangle (foals)/rectangle (mares) of the same colour.

Figure S2. Identity matrix for Stud I. Heatmap showing percent identity between pairs of equid herpesvirus 5 (EHV-5) partial gB sequences. Sequences are aligned along the x and y axes. Identity scores for each pairwise comparison are represented by coloured boxes with identity increasing from blue to red.

Figure S3. Identity matrix for Stud II. Heatmap showing percent identity between pairs of equid herpesvirus 5 (EHV-5) partial gB sequences. Sequences are aligned along the x and y axes. Identity scores for each pairwise comparison are represented by coloured boxes with identity increasing from blue to red.

Figure S4. Identity matrix for Stud III. Heatmap showing percent identity between pairs of equid herpesvirus 5 (EHV-5) partial gB sequences. Sequences are aligned along the x and y axes. Identity scores for each pairwise comparison are represented by coloured boxes with identity increasing from blue to red.
