# Supplementary figures and images for: Genetic Diversity of Equid Herpesvirus 5 in Temporal Samples from Mares and Their Foals at Three Polish National Studs
Source: Int J Mol Sci. 2025 Aug 27;26(17):8298. doi: 10.3390/ijms26178298 (PMC12428152; doi:10.3390/ijms26178298)

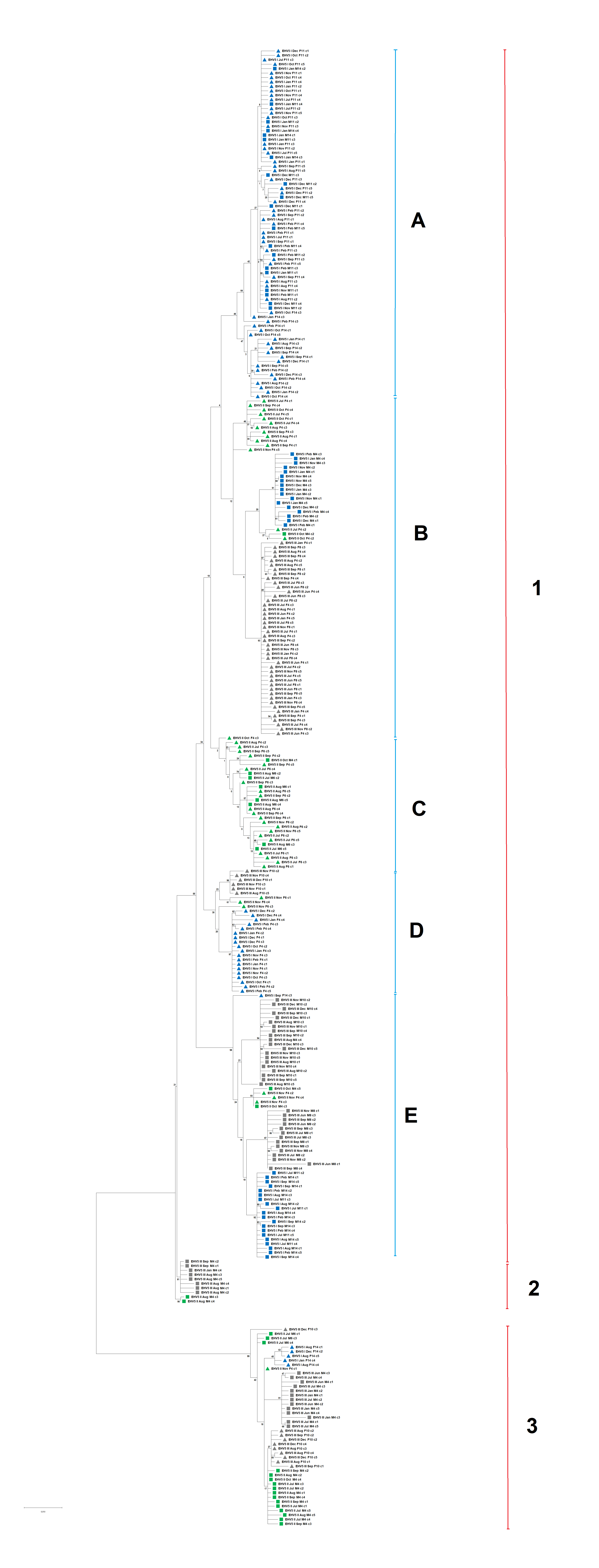

Supplement: Supplementary file 1 [file ijms-26-08298-s001.zip › Figure_S1.png]

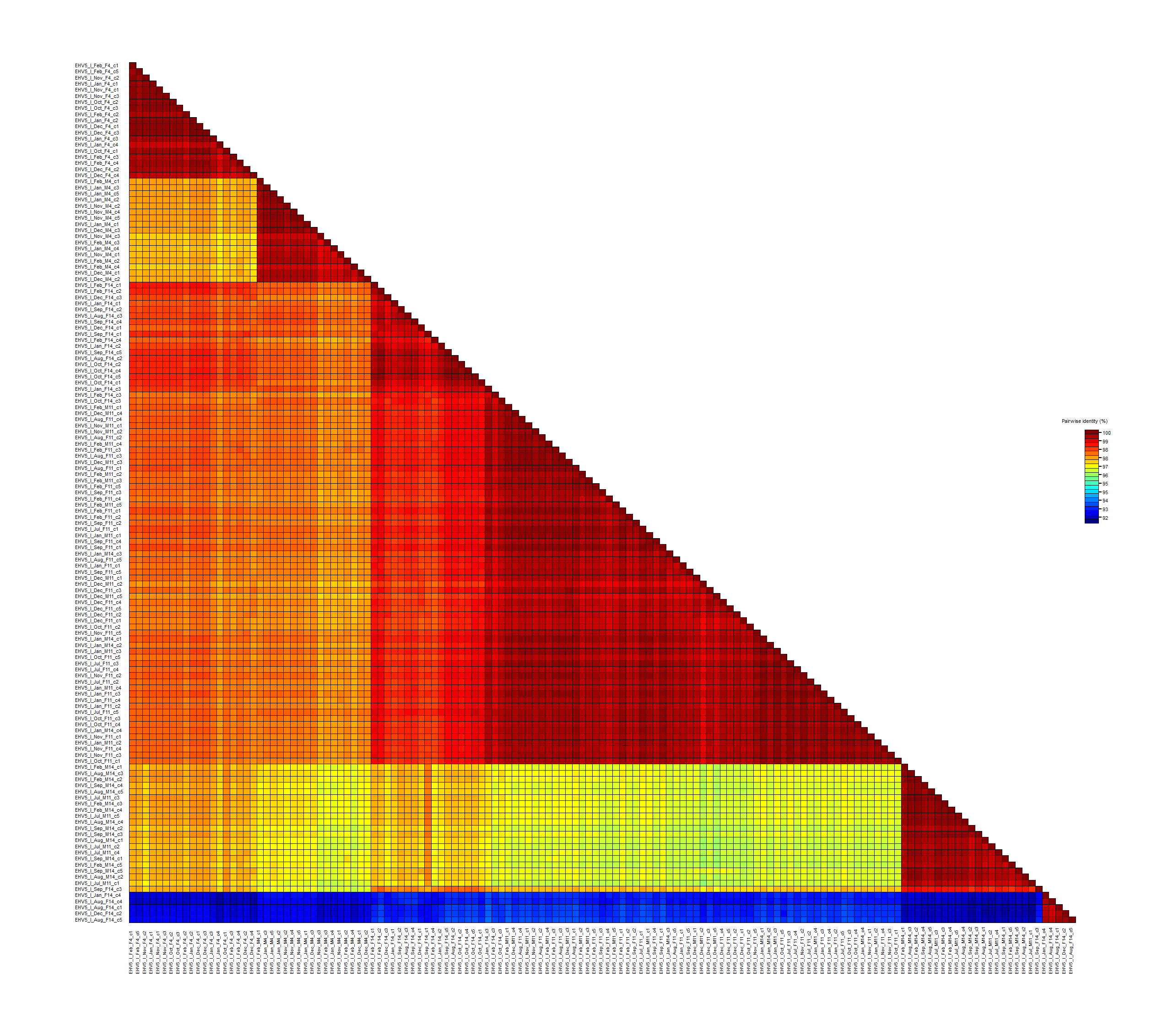

Supplement: Supplementary file 1 [file ijms-26-08298-s001.zip › Figure_S2.png]

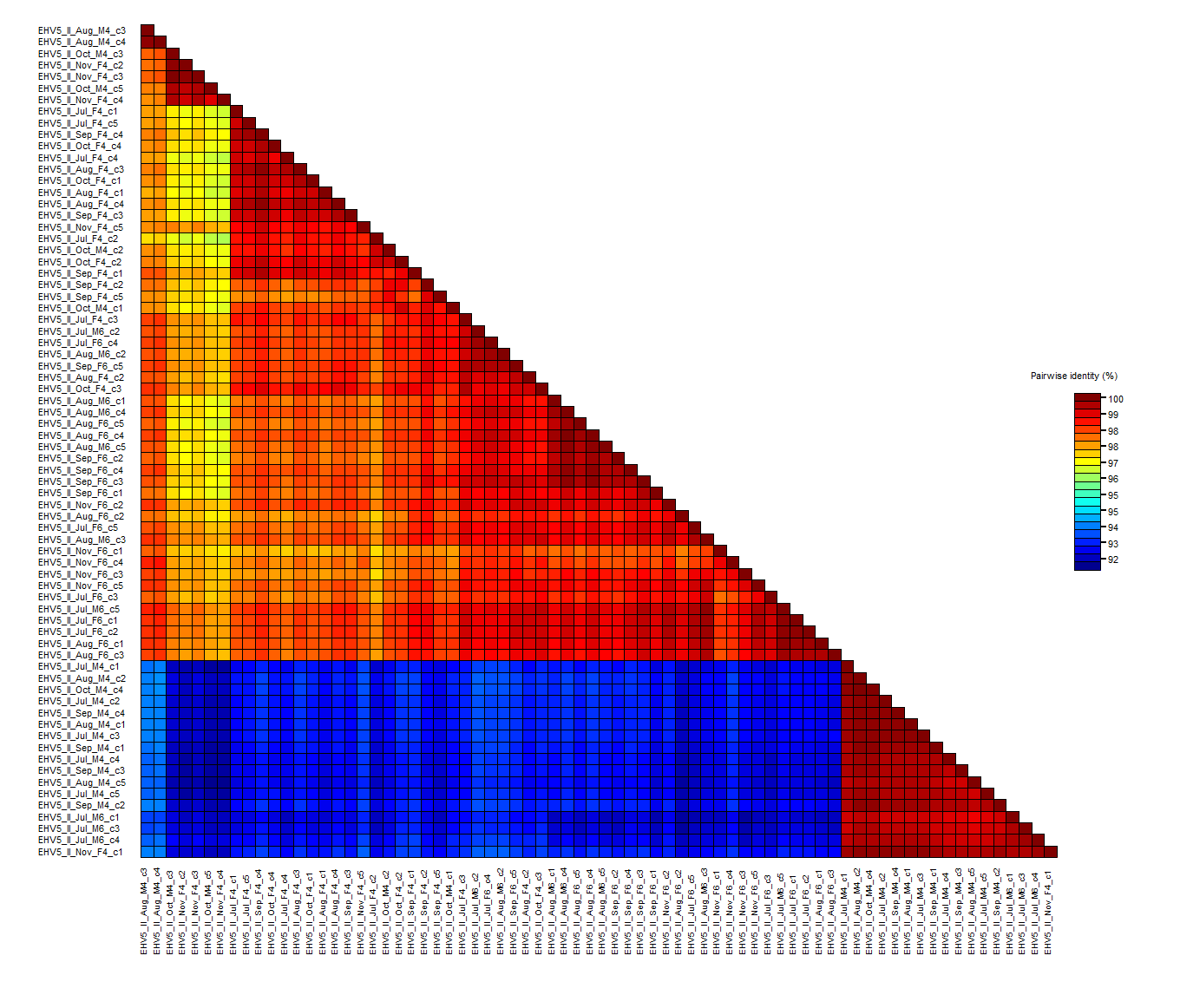

Supplement: Supplementary file 1 [file ijms-26-08298-s001.zip › Figure_S3.png]

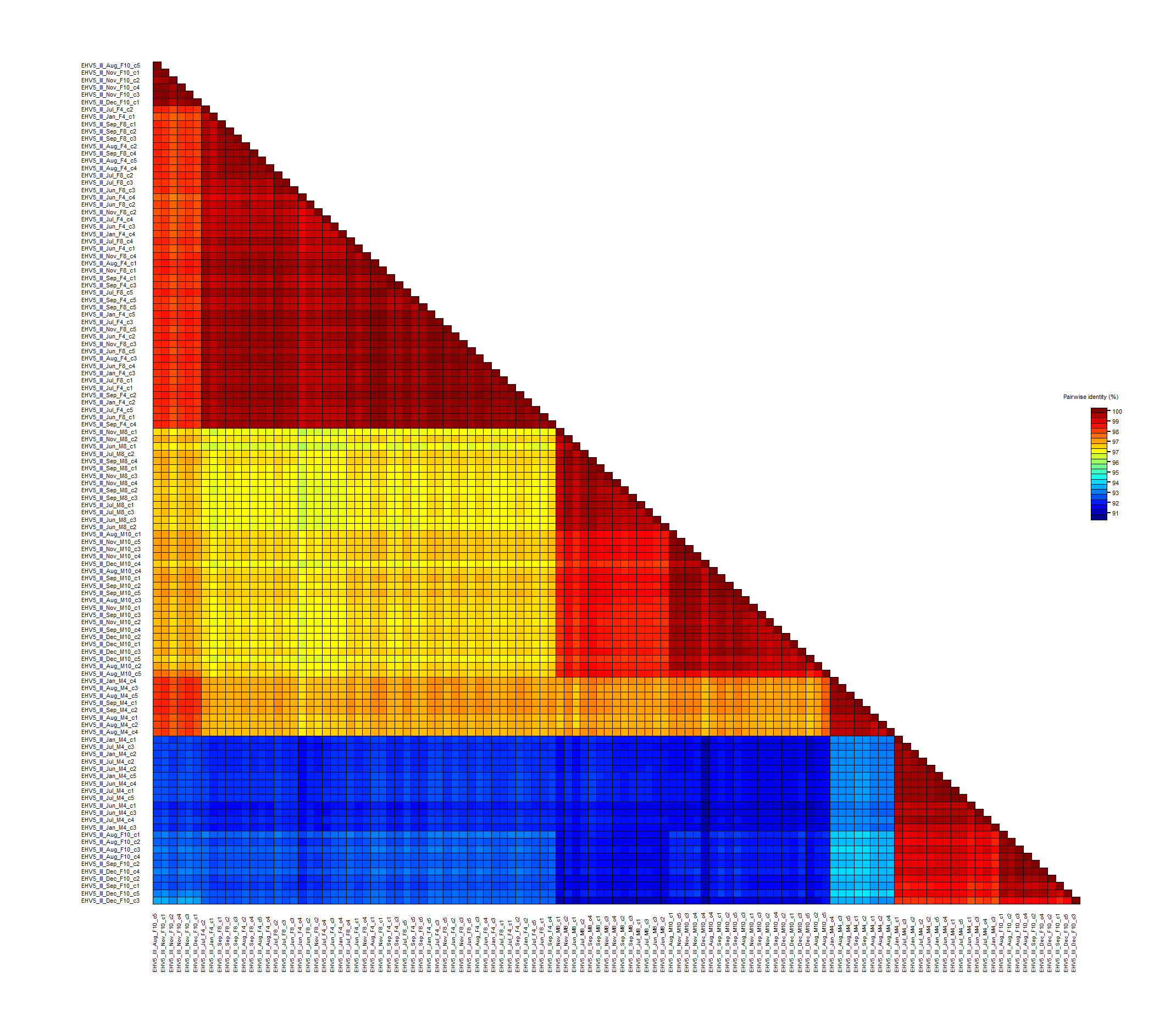

Supplement: Supplementary file 1 [file ijms-26-08298-s001.zip › Figure_S4.png]
